# Supplementary figures and images for: Long divergent haplotypes introgressed from wild sheep are associated with distinct morphological and adaptive characteristics in domestic sheep
Source: PLoS Genet. 2023 Feb 23;19(2):e1010615. doi: 10.1371/journal.pgen.1010615 (PMC9949681; doi:10.1371/journal.pgen.1010615)

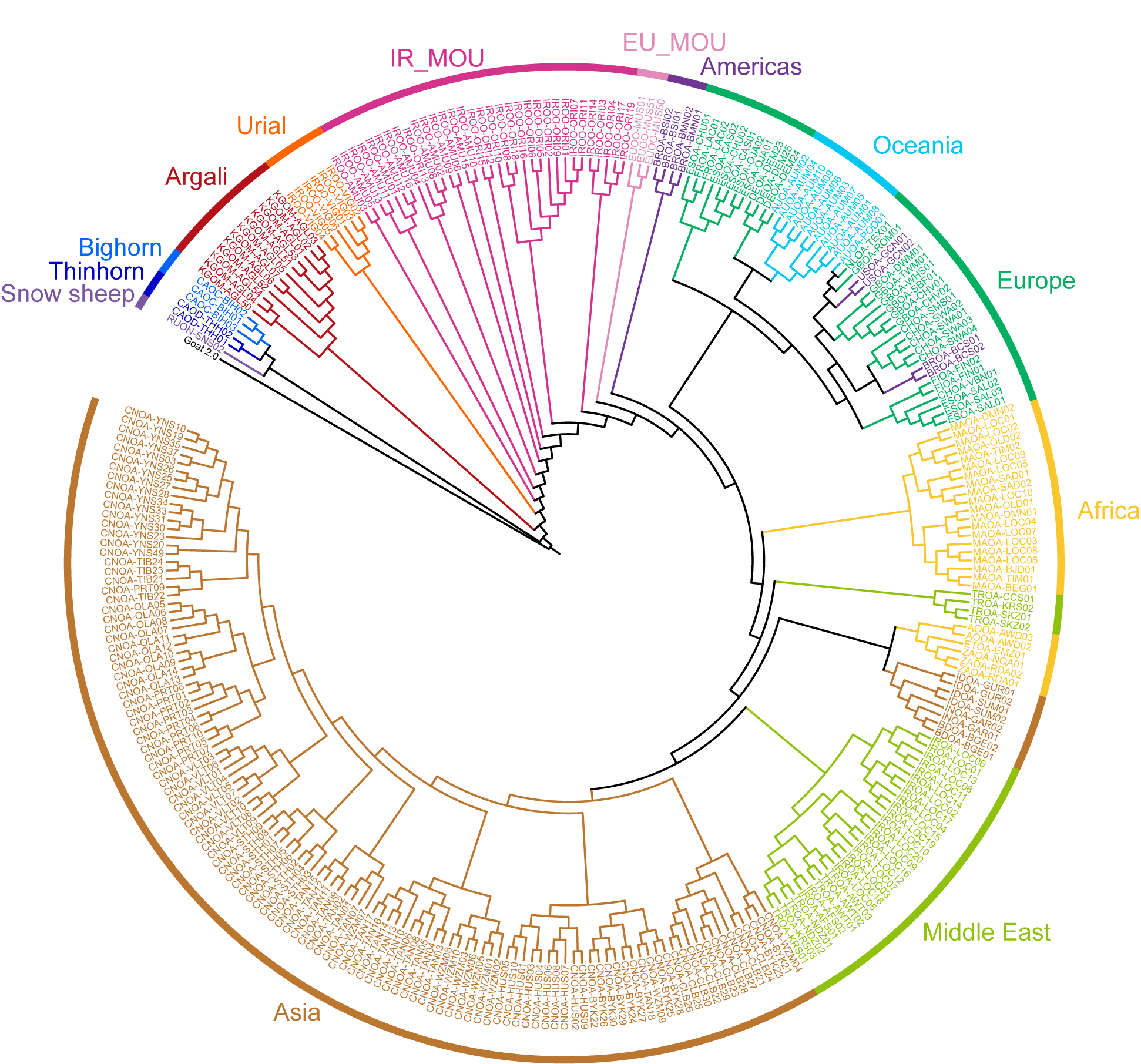

Supplement: S1 Fig — Goat was used as outgroup. (TIF) [file pgen.1010615.s001.tif]

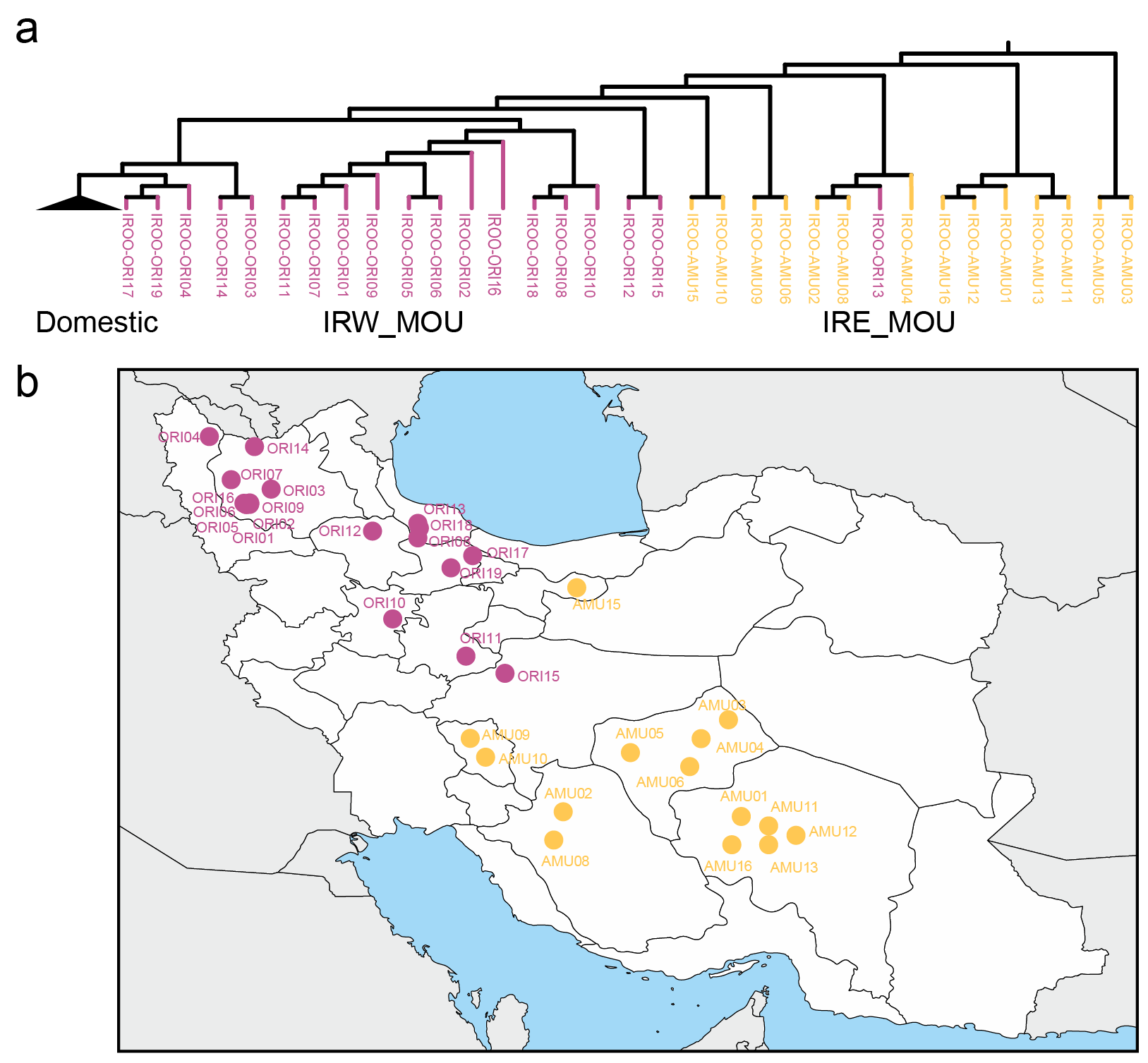

Supplement: S2 Fig — The map shows the geographic distribution of 33 Iranian mouflon samples, which can be divided into two groups according to their geographical distribution, western Iran (IRW_MOU) and eastern Iran (IRE_MOU). https://d-maps.com/carte.php?num_car=5494&lang=zh (TIF) [file pgen.1010615.s002.tif]

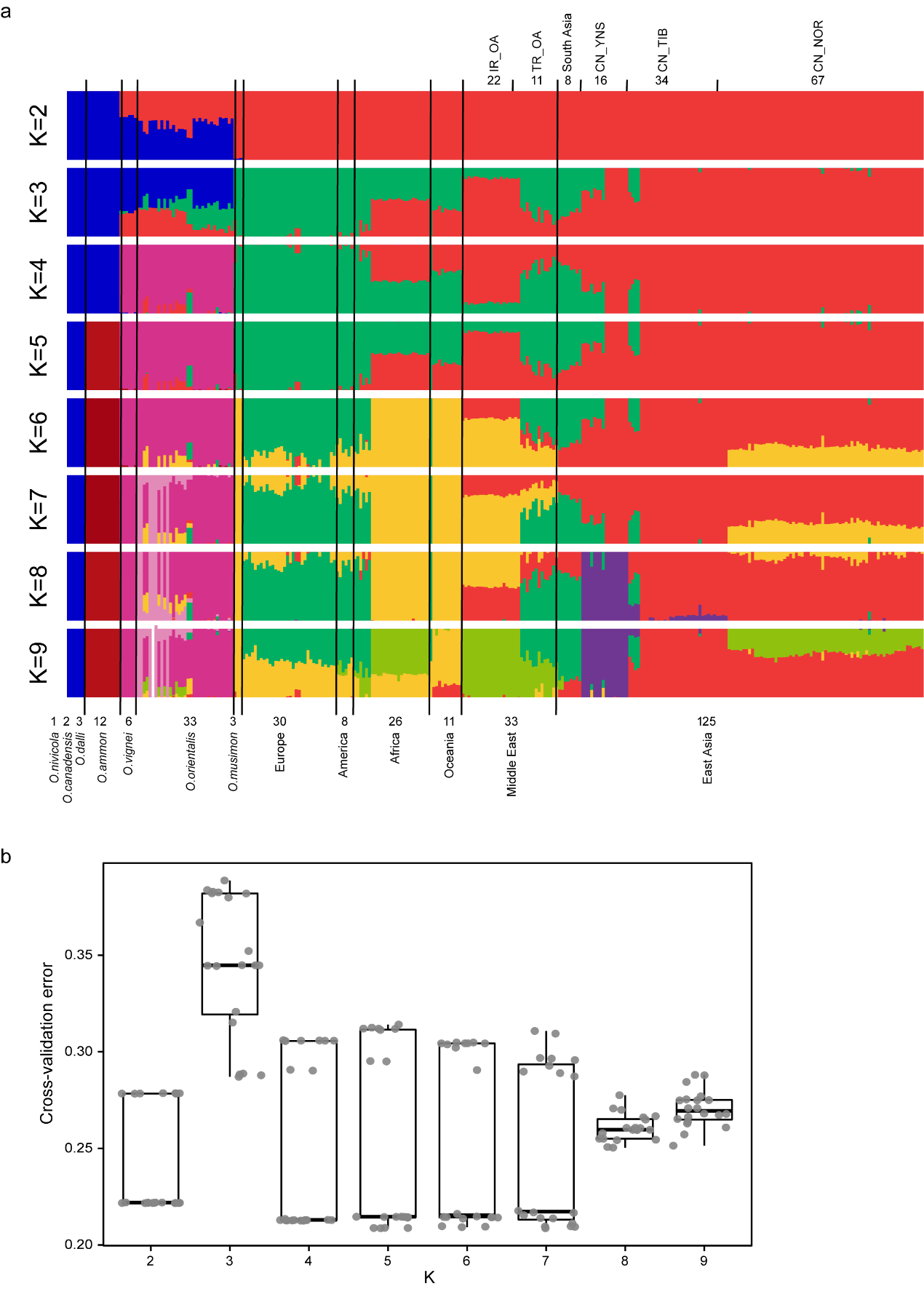

Supplement: S3 Fig — (a) ADMIXTURE results for k = 2 to k = 9. For each k value, the run with the lowest cross validation (CV) error out of 20 replicates is plotted. The number of samples and population names are listed both at the top and bottom. (b) CV error for varying k in the ADMIXTURE analysis. (TIF) [file pgen.1010615.s003.tif]

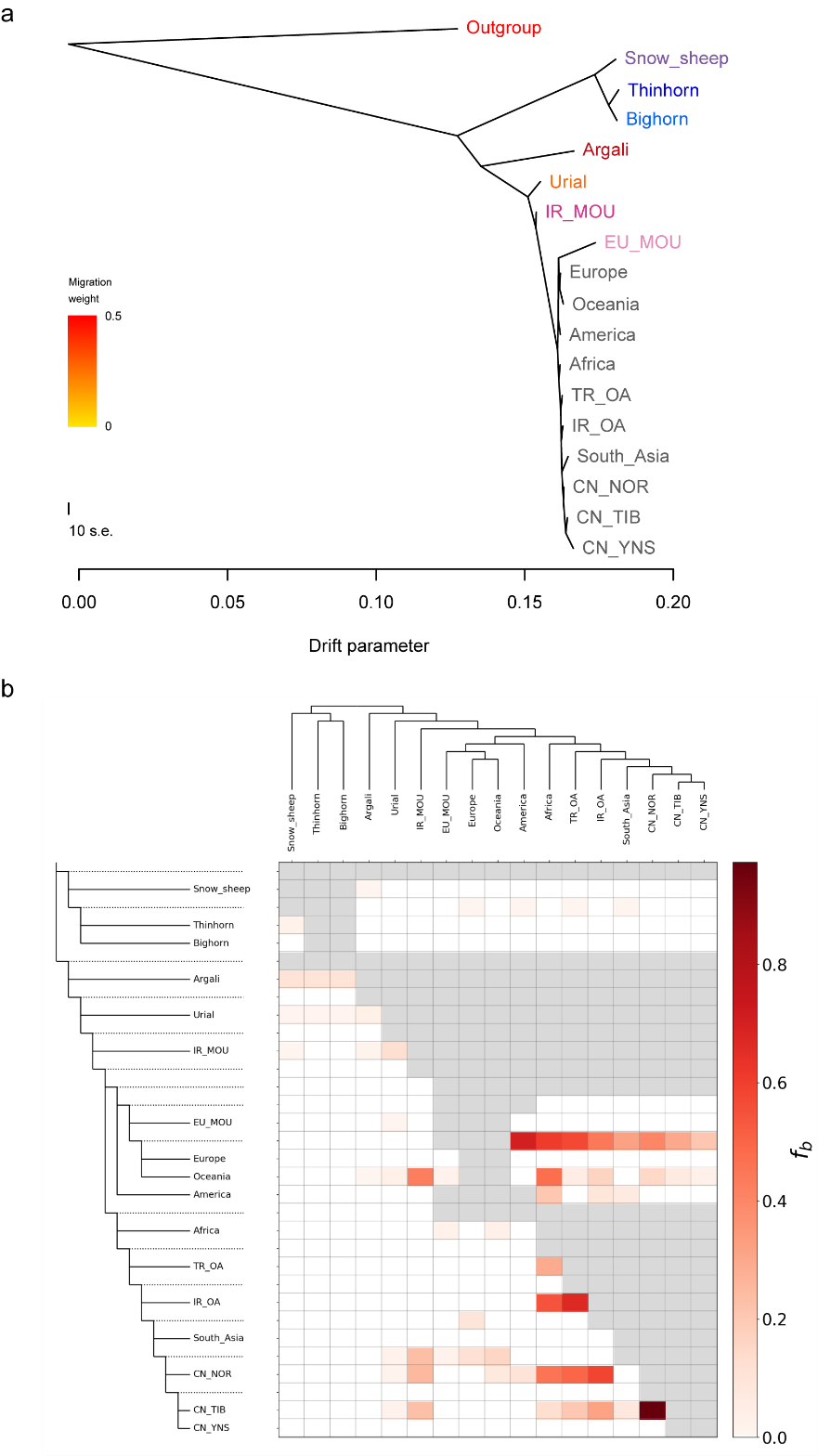

Supplement: S4 Fig — (a) The species/population tree constructed by Treemix ignored gene flow, and goat was used as outgroup. (b) Results of Fbranch in Dsuite for wild sheep species and different domestic sheep populations. The species/populations tree is shown along the left and upper sides, and the tree on the y axis is displayed in “expanded” form. The color-block in the matrix refer to the excess allele sharing between the branch identified on the expanded tree on the y axis and the species/populations on the x-axis. The darker the color means the higher ratio in allele sharing, the lighter the ratio is lower. (TIF) [file pgen.1010615.s004.tif]

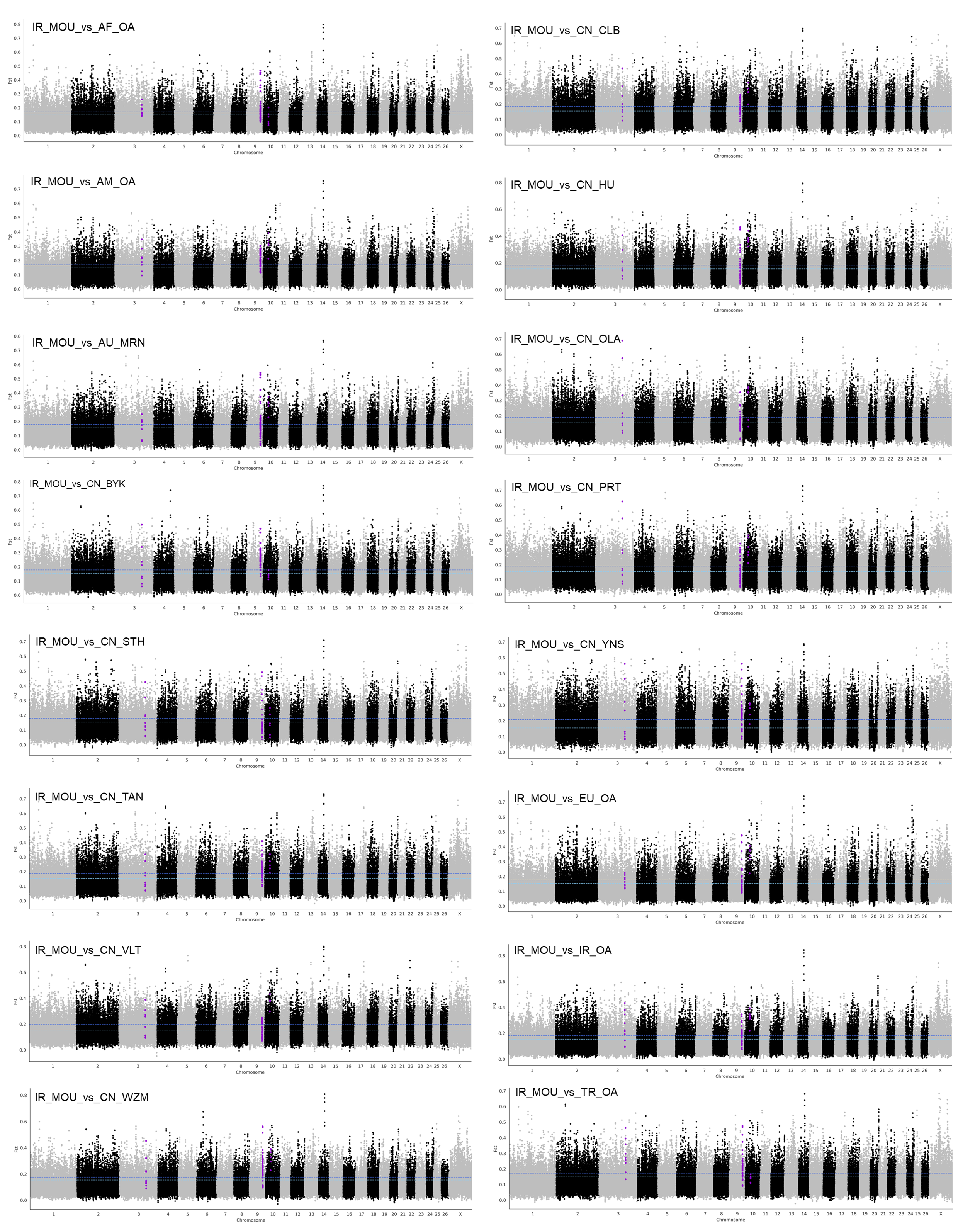

Supplement: S5 Fig — In addition, MSRB3(chromosome 3), VPS13B (chromosome 9) and RXFP2 (chromosome 10) were also highlight in purple. The blue dashed lines show the FST threshold (P <0.001, Z test), and the shallow blue dashed lines show the permutation threshold with 100 bootstrap. (TIF) [file pgen.1010615.s005.tif]

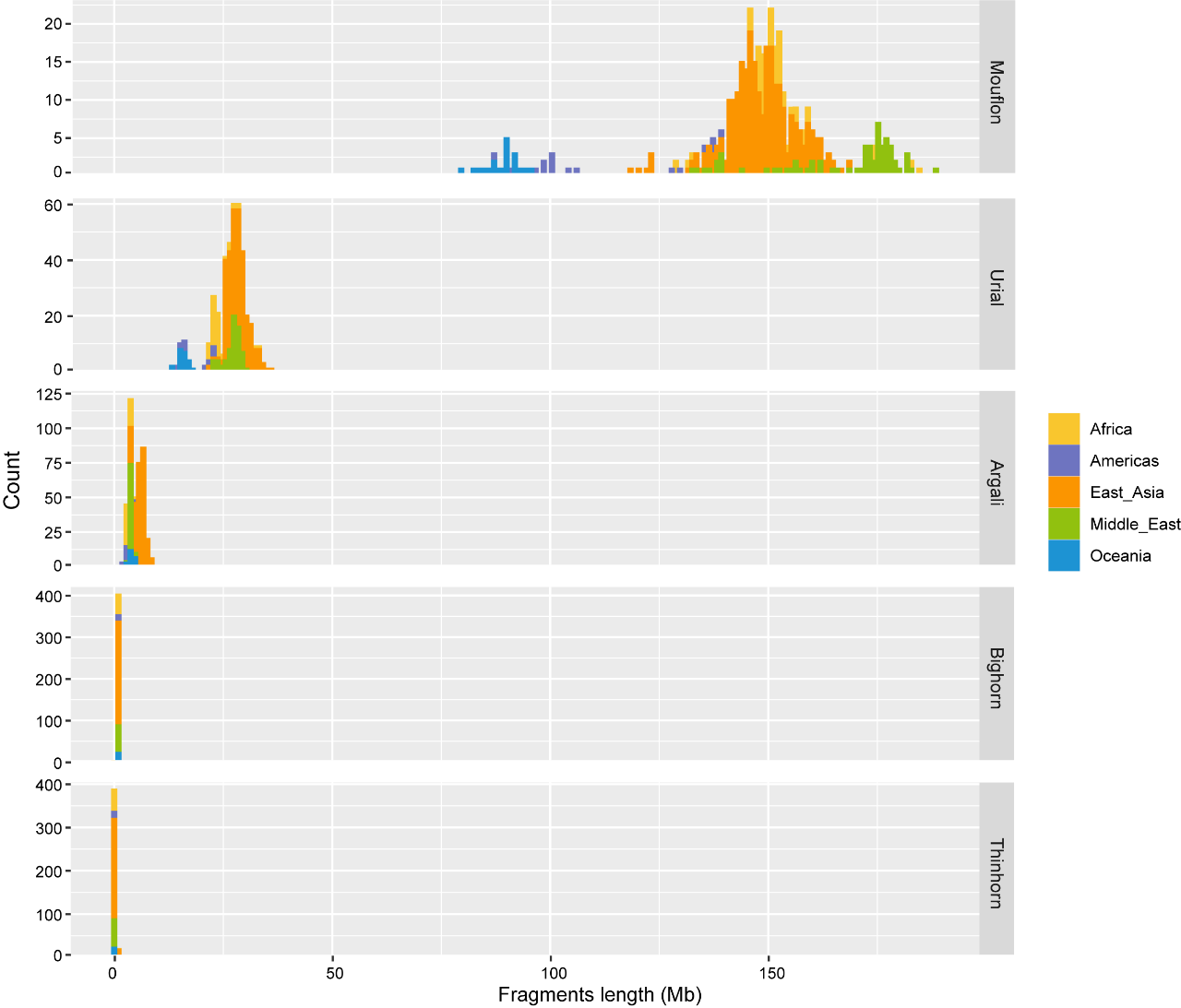

Supplement: S6 Fig — Incomplete lineage sorting (ILS) is precluded. Different color means domestic sheep populations originated from divergent region. (TIF) [file pgen.1010615.s006.tif]

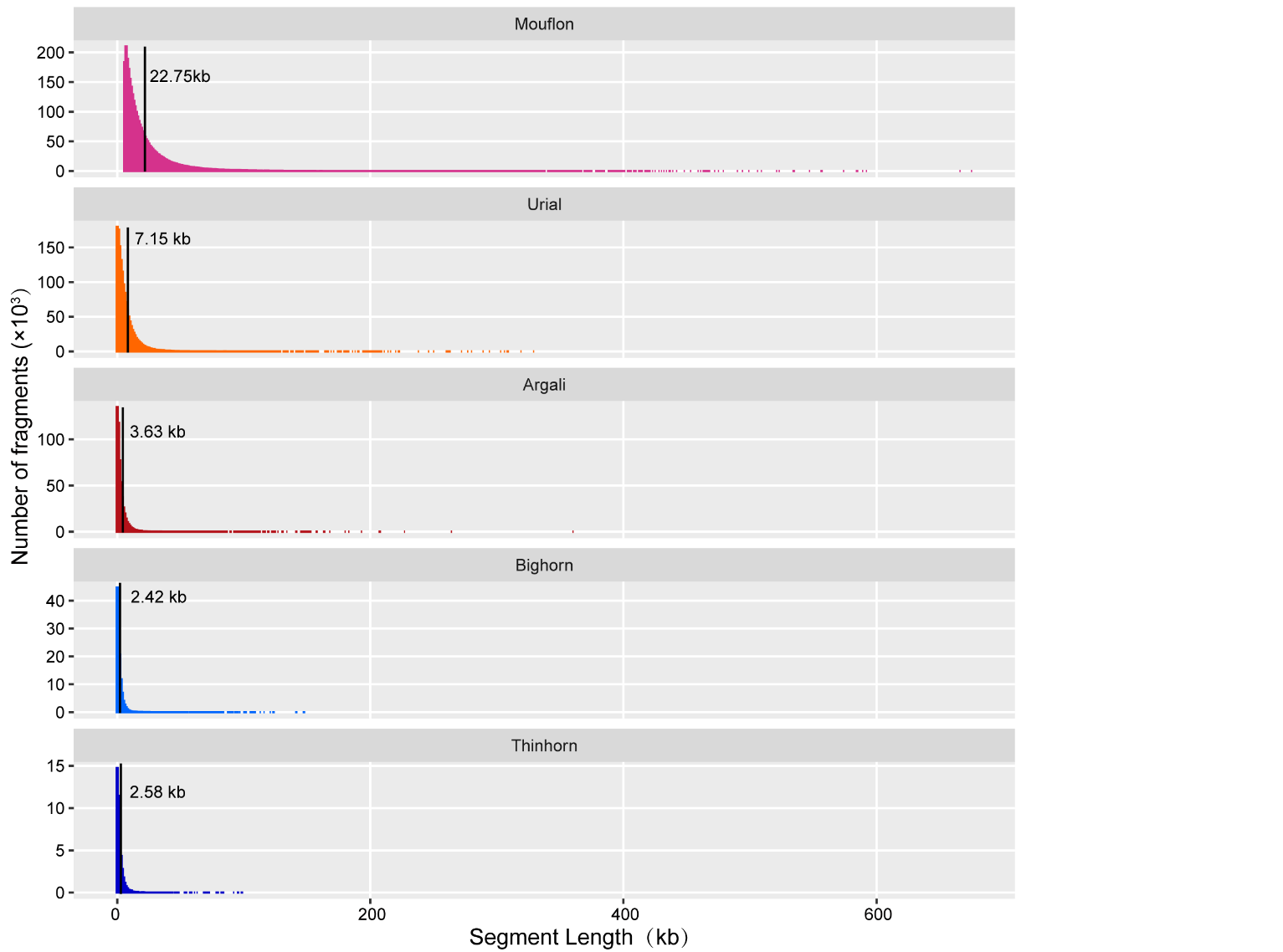

Supplement: S7 Fig — The vertical black lines indicate the mean segments length. (TIF) [file pgen.1010615.s007.tif]

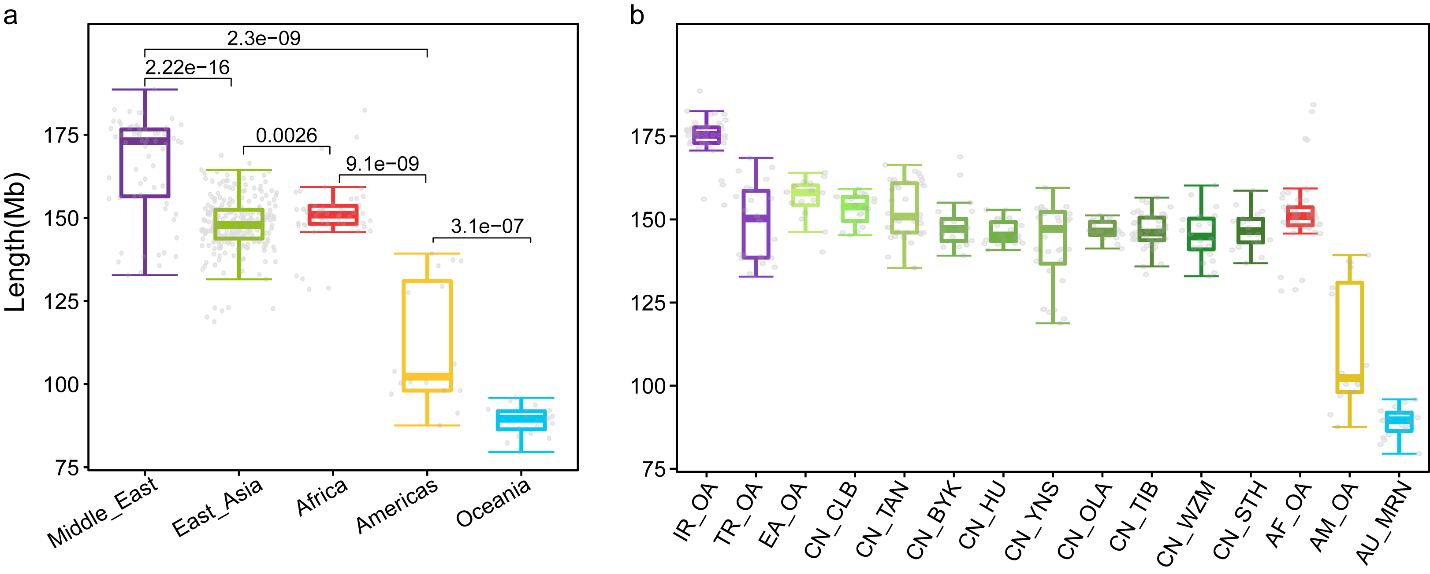

Supplement: S8 Fig — (a) The indicated P values are based on t-test. (b) Different colors represent different geographic regions displayed in a. (TIF) [file pgen.1010615.s008.tif]

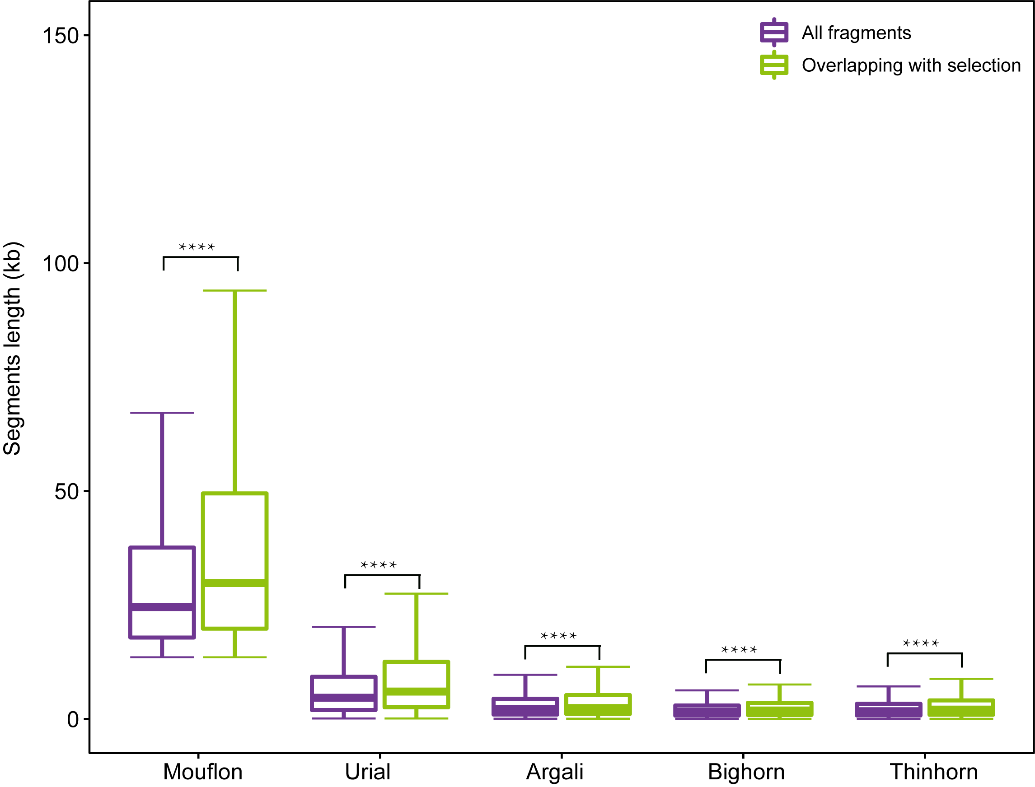

Supplement: S9 Fig — Purple: all introgressed fragments; green: segments with FST values above the threshold. The **** represents significant difference (P < 0.05). (TIF) [file pgen.1010615.s009.tif]

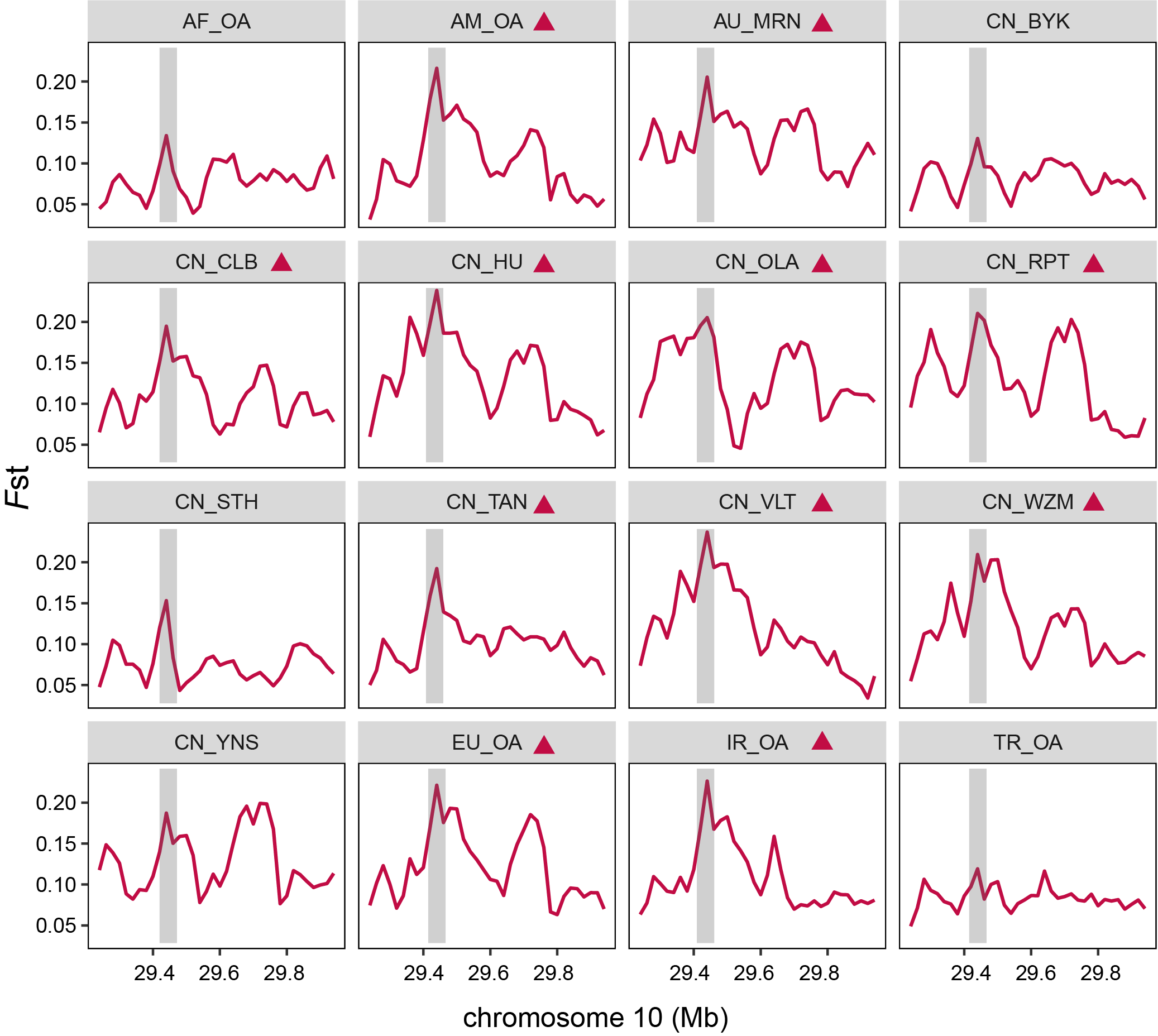

Supplement: S11 Fig — The FST was calculated in 50-kb sliding window with 20-kb step size. Triangles beside the population labels indicate that the population showed selective signals (FST top 1%) in this region. The grey box means the location of windows showing selective signal. (TIF) [file pgen.1010615.s011.tif]

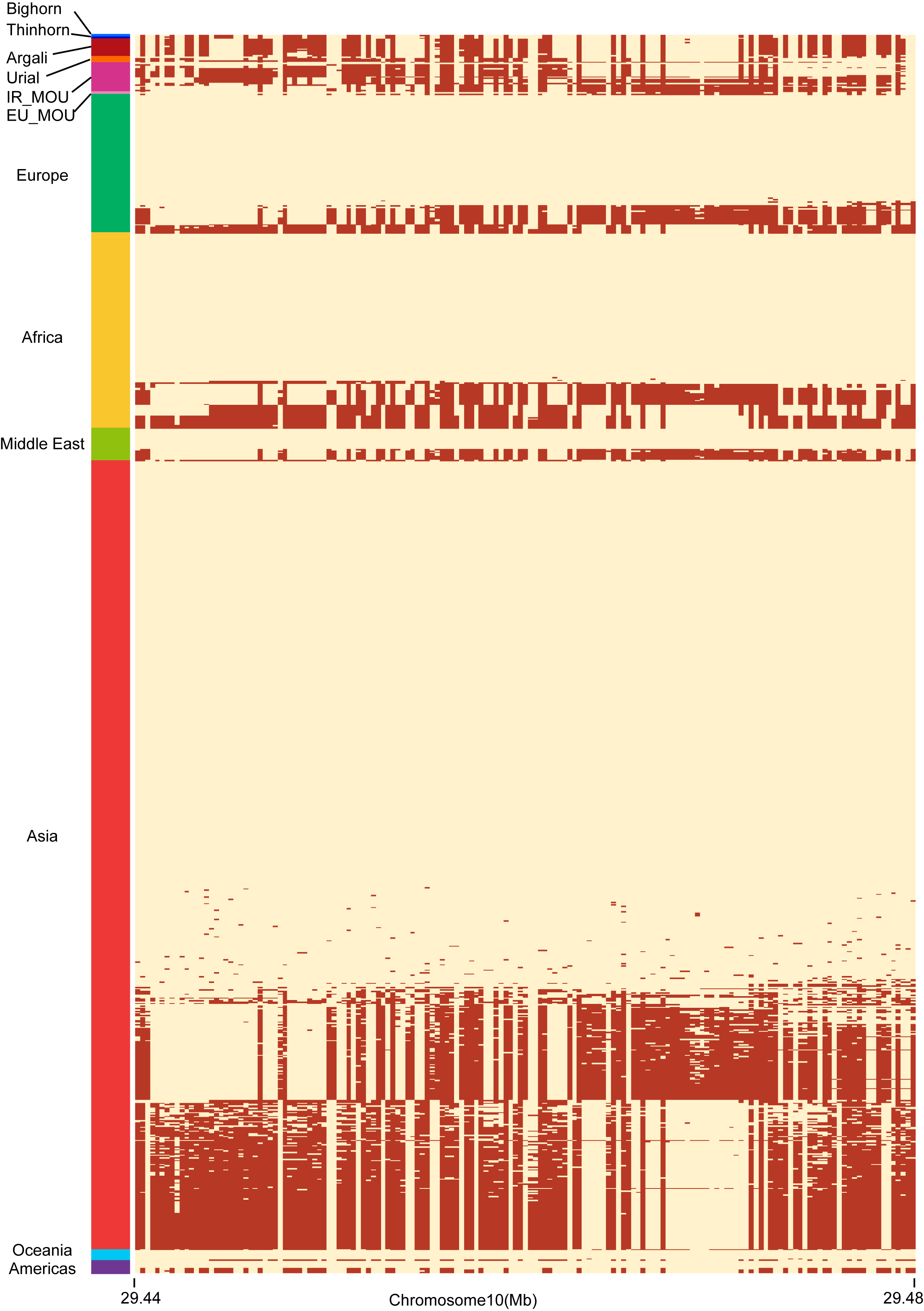

Supplement: S12 Fig — Each column represents a SNP variant, and each row represents a phased haplotype. Yellow predicted alleles identical to reference genome; red predicted alleles differ to reference genome. (TIF) [file pgen.1010615.s012.tif]

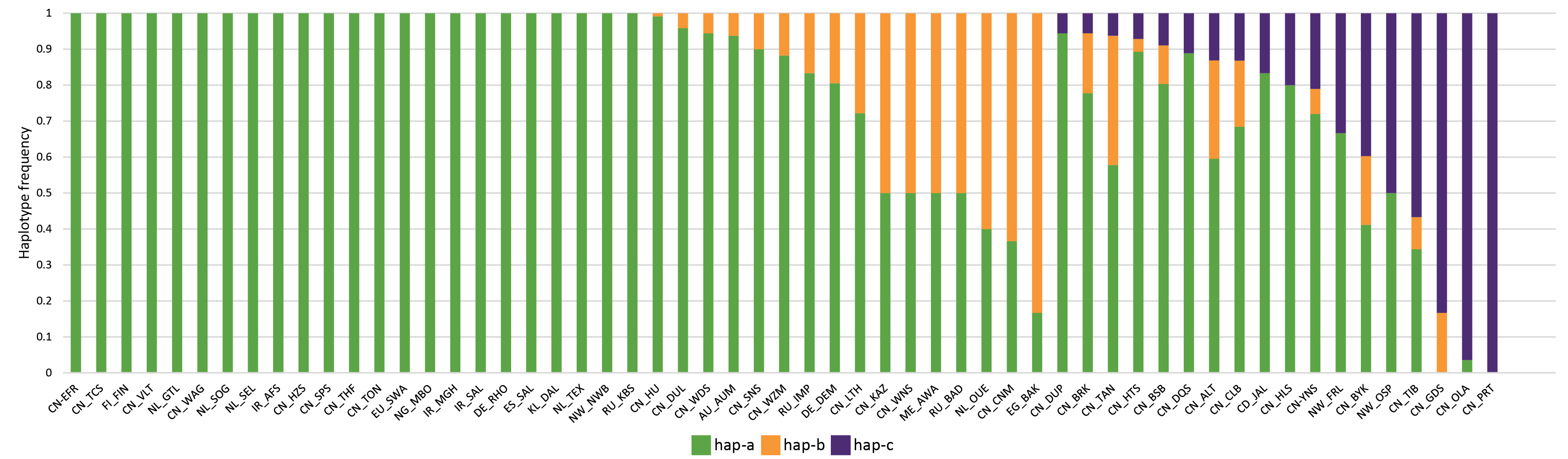

Supplement: S13 Fig — (TIF) [file pgen.1010615.s013.tif]

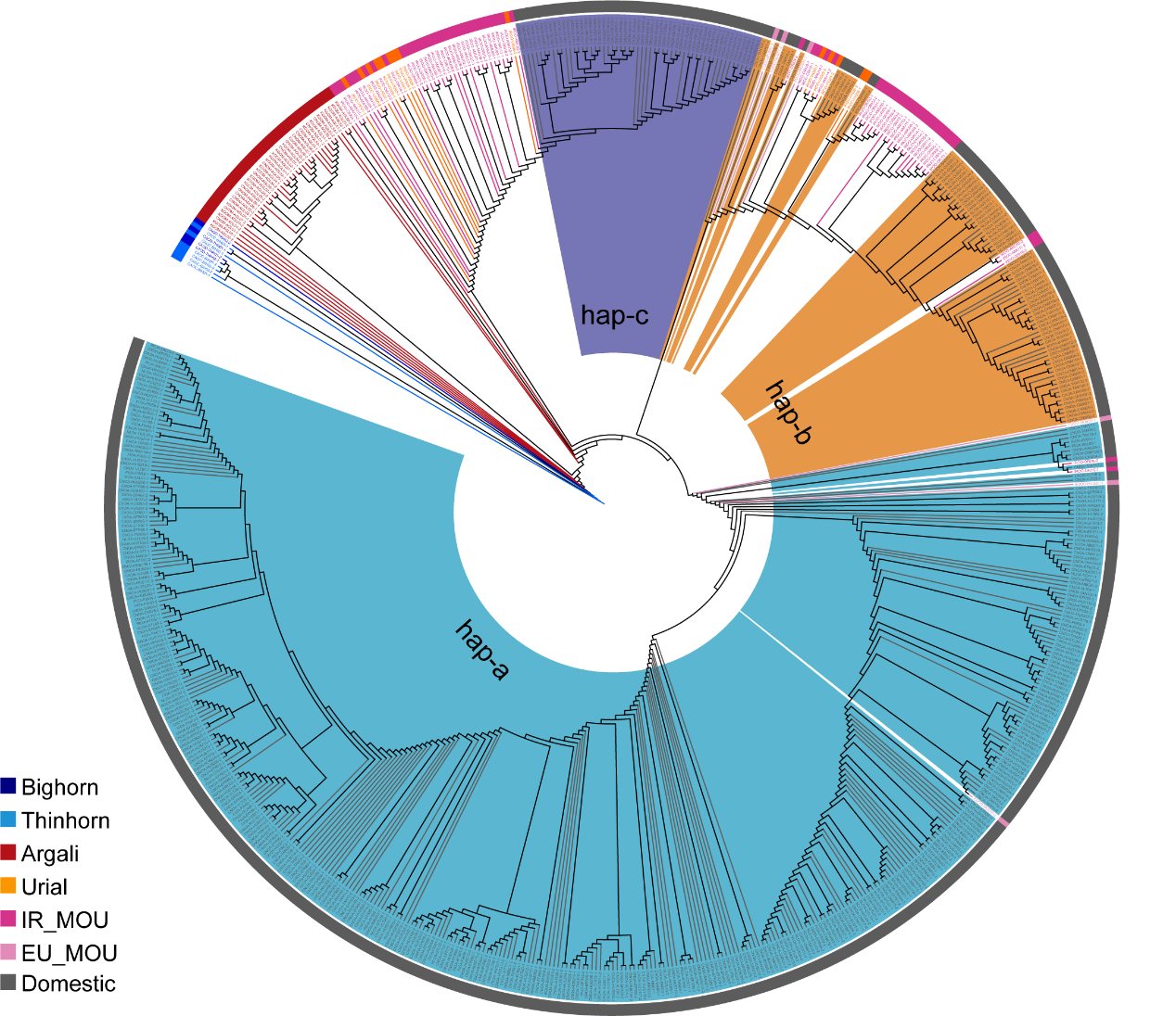

Supplement: S14 Fig — The ML tree was built using 221 SNPs with minor allele frequency (MAF) > 0.05 from 29,435,112 to 29,481,215 on chromosome 10 with 100 bootstraps. (TIF) [file pgen.1010615.s014.tif]

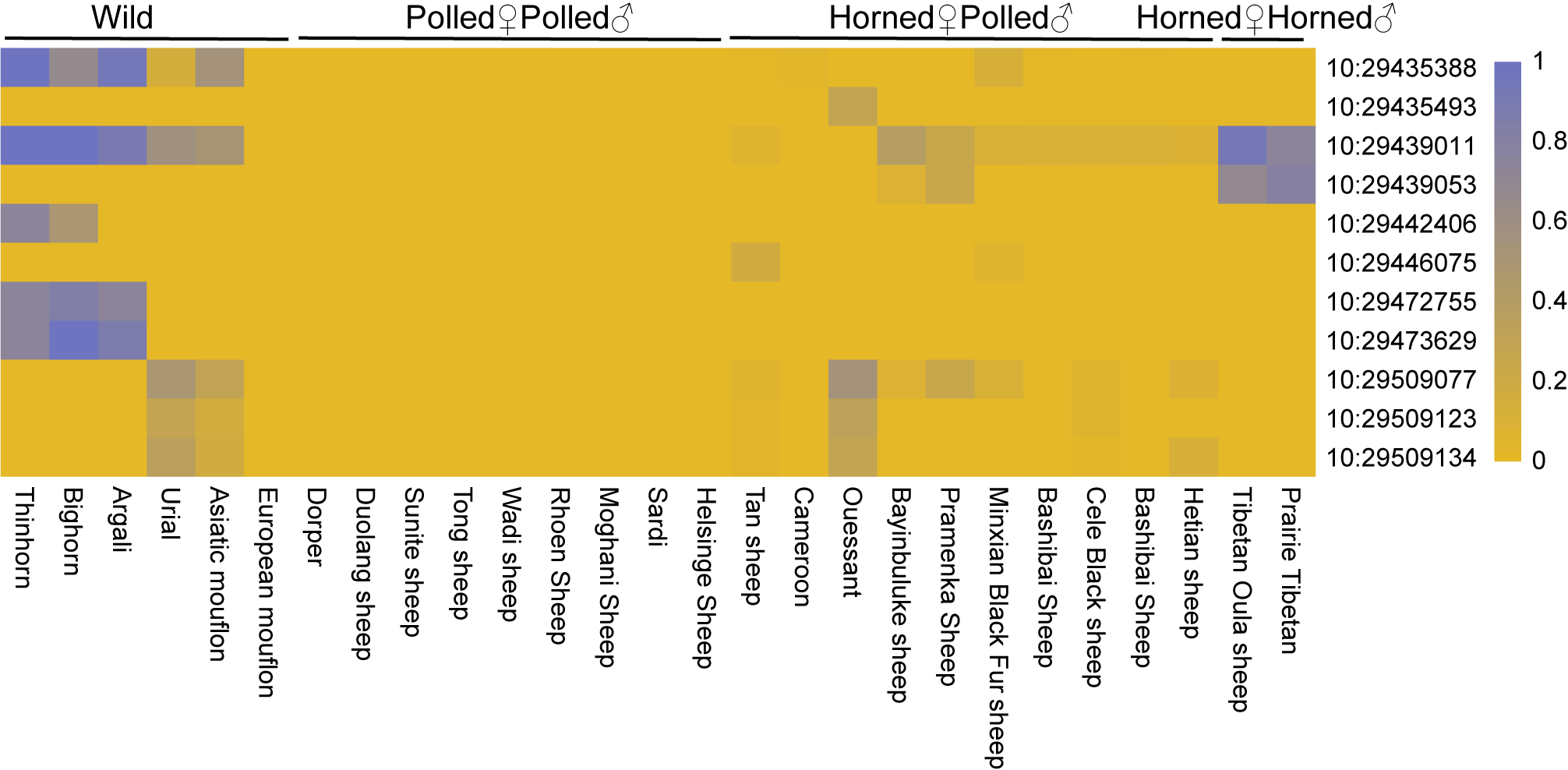

Supplement: S15 Fig — The species or population names are listed at the bottom, the corresponding horn types of domestic sheep are showed on the top. (TIF) [file pgen.1010615.s015.tif]

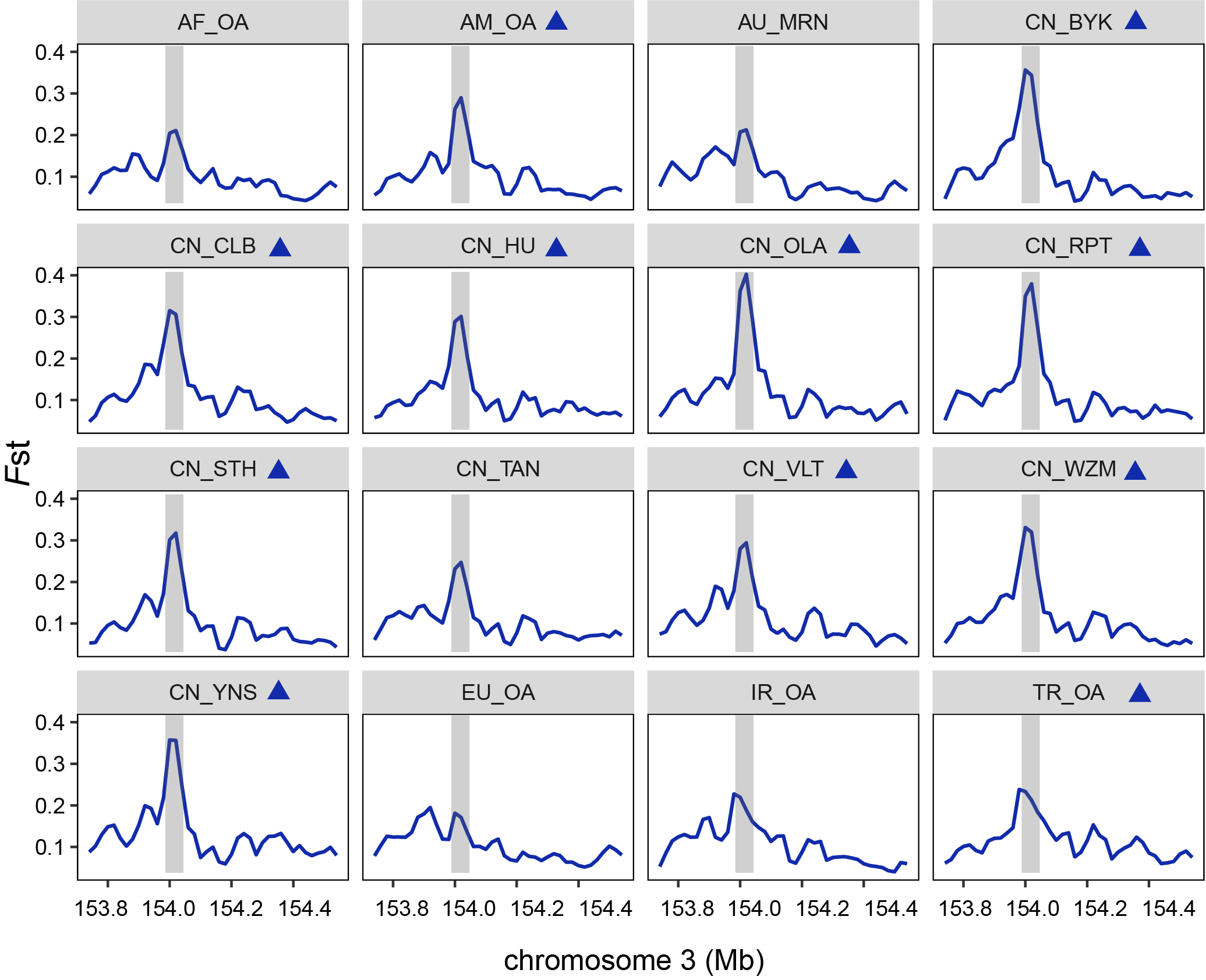

Supplement: S16 Fig — The FST was calculated in 50-kb sliding window with 20-kb step size. Triangles beside the population labels indicate that the population showed selective signals (FST top 1%) in MSRB3. The grey box means the location of windows showing selective signal. (TIF) [file pgen.1010615.s016.tif]

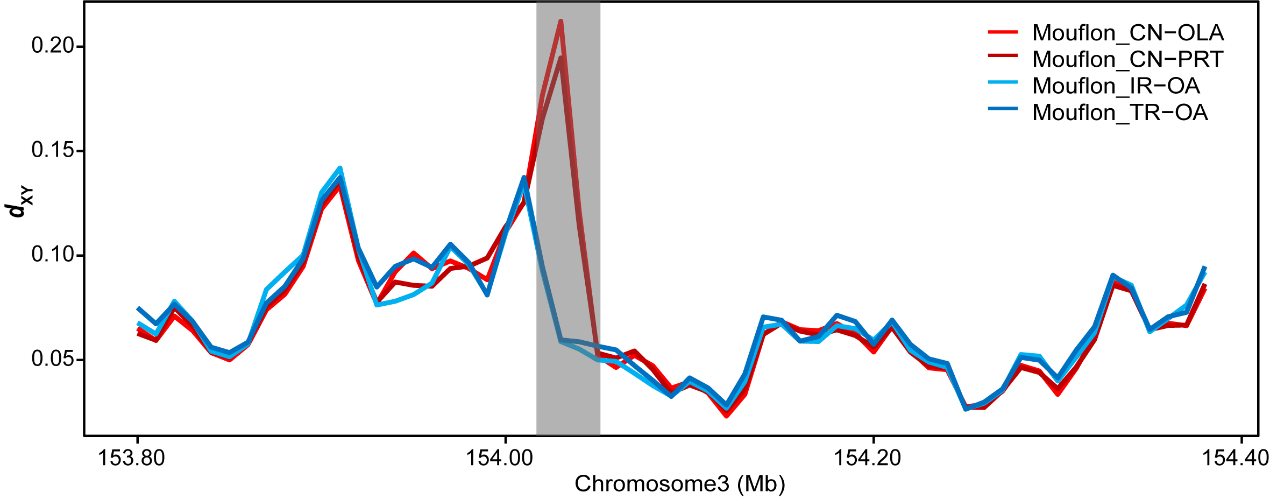

Supplement: S17 Fig — Introgressed region (chr3: 154,030,492–154,062,195) are gray-shaded. The dXY values between Iranian mouflon and Oula (CN-OLA)/ Prairie Tibetan (CN-PRT) showed a marked increase in the introgressed region, compared with Iran (IR-OA) and Turkey (TR-OA) sheep. The dXY values are calculated in 50-kb sliding window with 20-kb step size. (TIF) [file pgen.1010615.s017.tif]

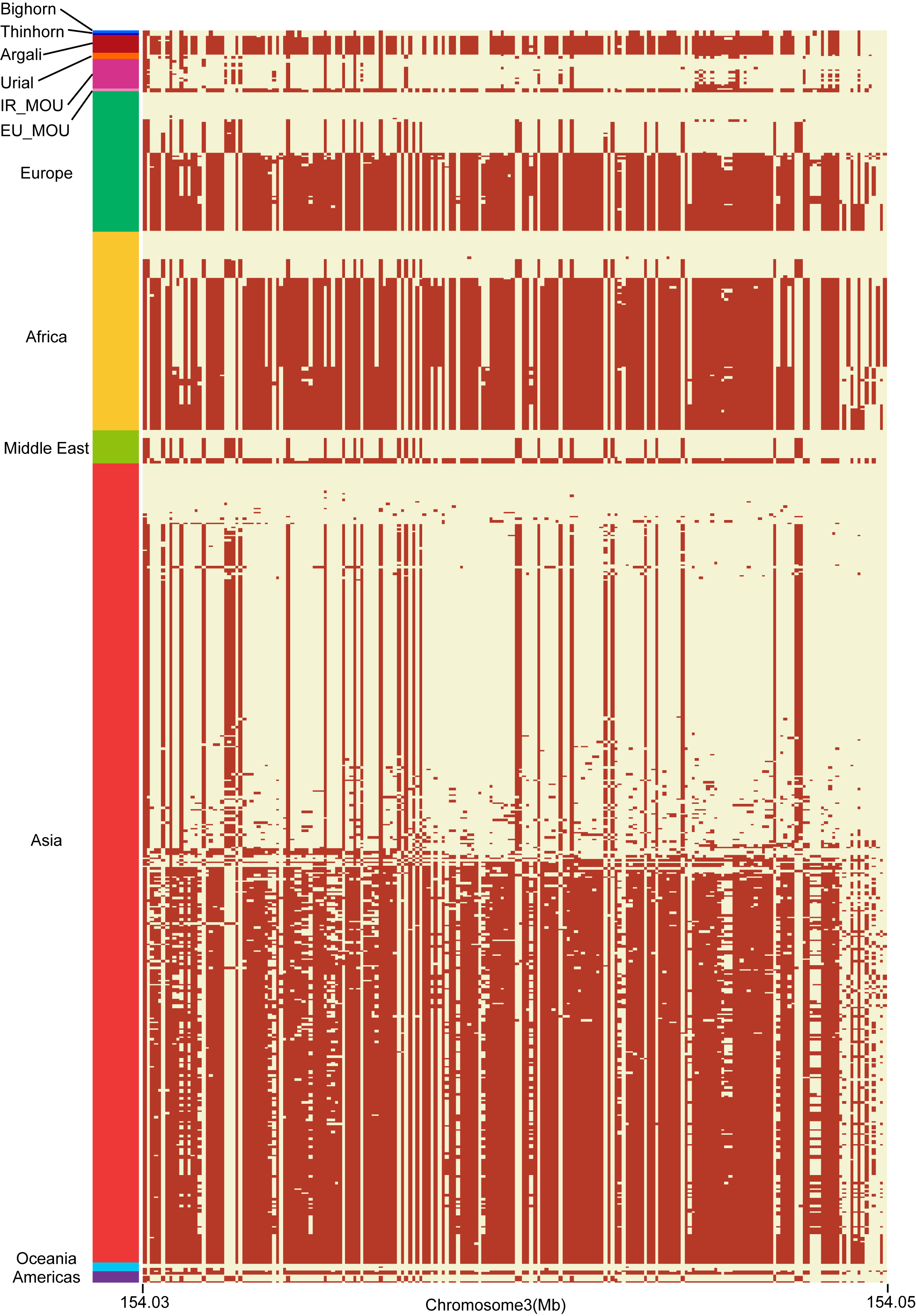

Supplement: S18 Fig — Each column represents a SNP variant, and each row represents a phased haplotype. Yellow predicted alleles identical to reference genome; red predicted alleles differ to reference genome. (TIF) [file pgen.1010615.s018.tif]

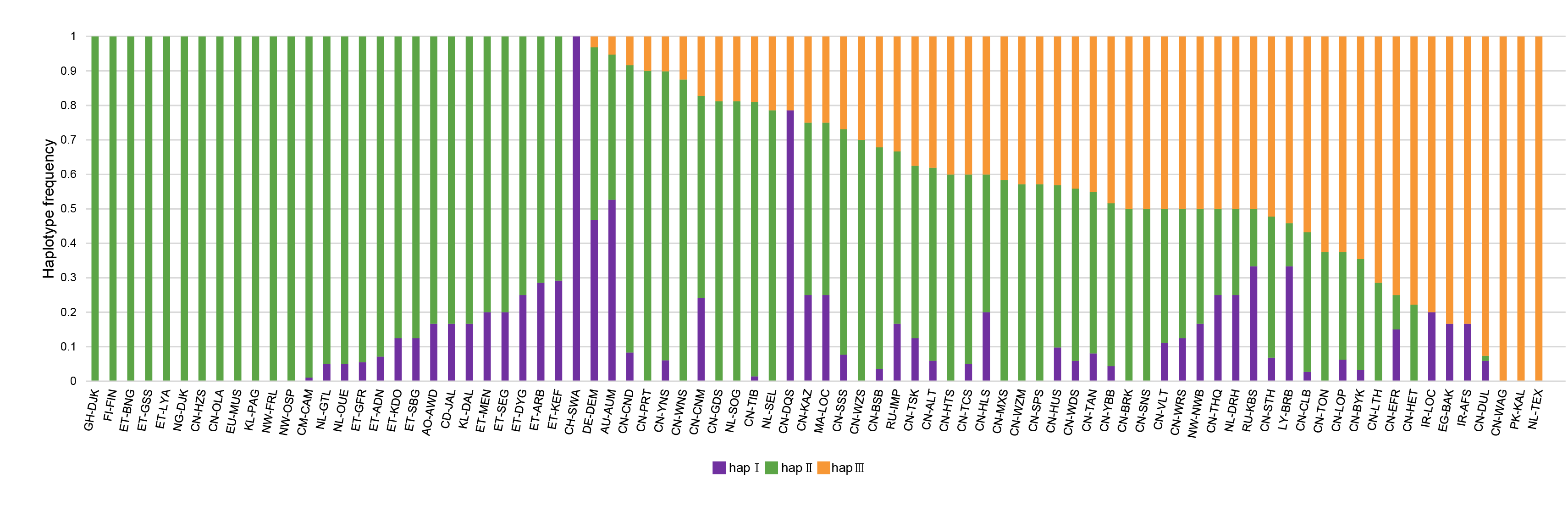

Supplement: S19 Fig — (TIF) [file pgen.1010615.s019.tif]

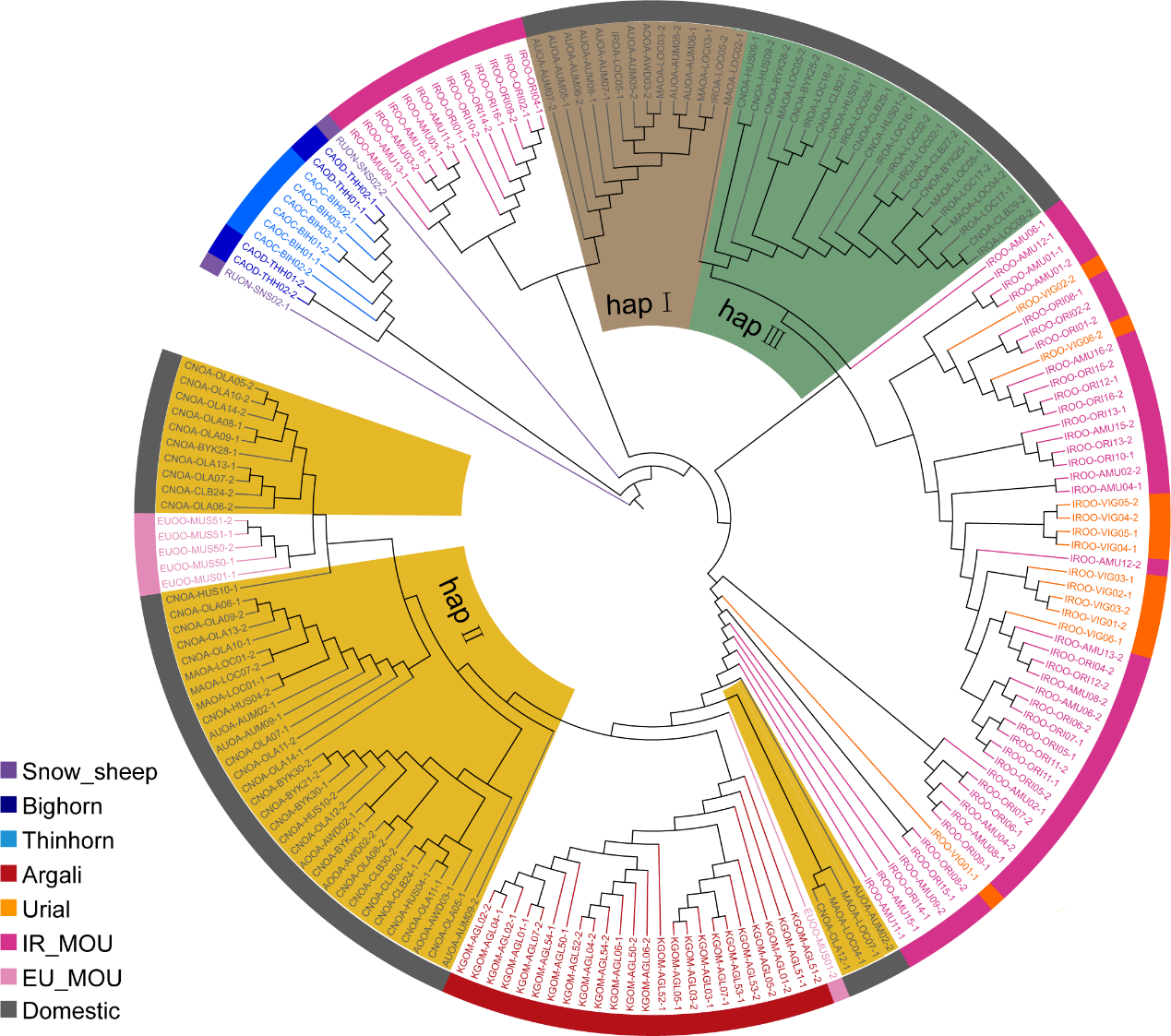

Supplement: S20 Fig — The ML tree was built using 327 SNPs with minor allele frequency (MAF > 0.05) from 154,030,492 to 154,053,023 on chromosome 3 by 100 bootstraps. (TIF) [file pgen.1010615.s020.tif]

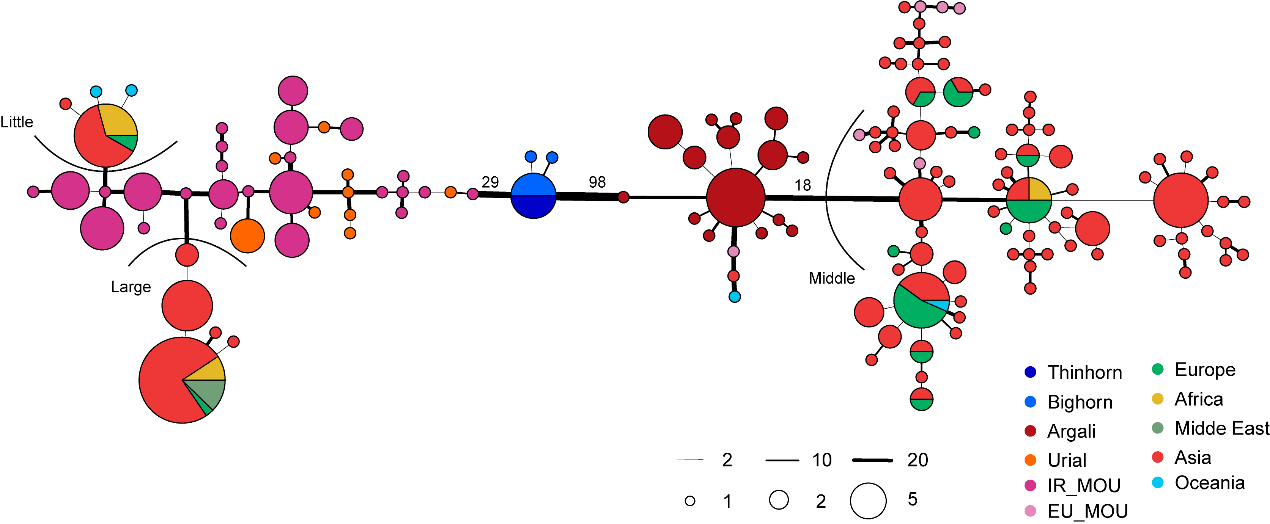

Supplement: S21 Fig — Different color shows different wild species or different regional sources of domestic sheep. Three major haplotypes and some haplotypes with lower frequencies in domestic sheep were identified. The R software package PEGAS were used to generate the network. (TIF) [file pgen.1010615.s021.tif]

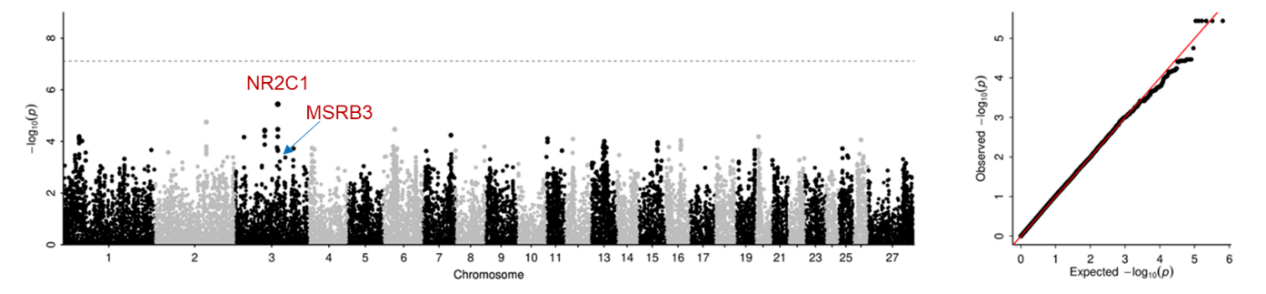

Supplement: S22 Fig — The gray horizontal dashed line indicates the significance threshold of the GWAS (P = 7.72e-08). (TIF) [file pgen.1010615.s022.tif]

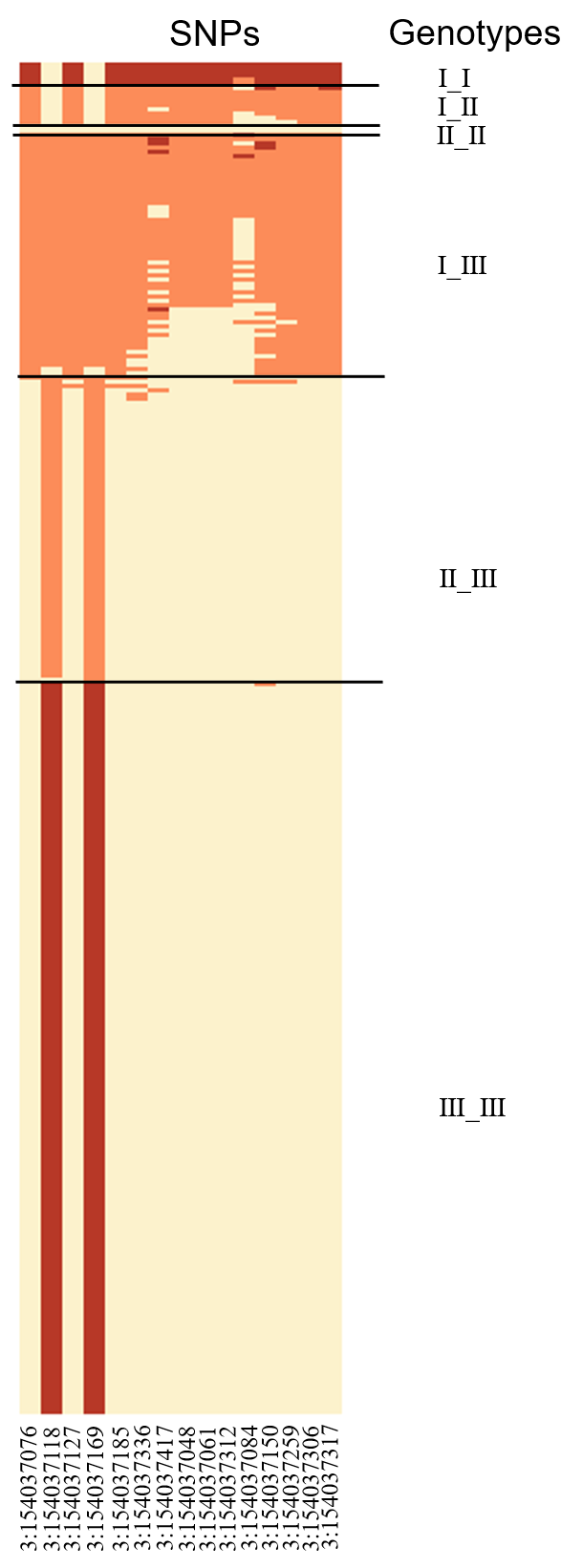

Supplement: S23 Fig — These SNPs are genotyped by target sequencing. Each column indicates a variation significantly associated with ear width, III_III refers to homozygous hapIII; other genotypes are denoted accordingly. Homozygous refence, heterozygous variant and homozygous variant are indicated in light beige, orange and brick red, respectively. (TIF) [file pgen.1010615.s023.tif]

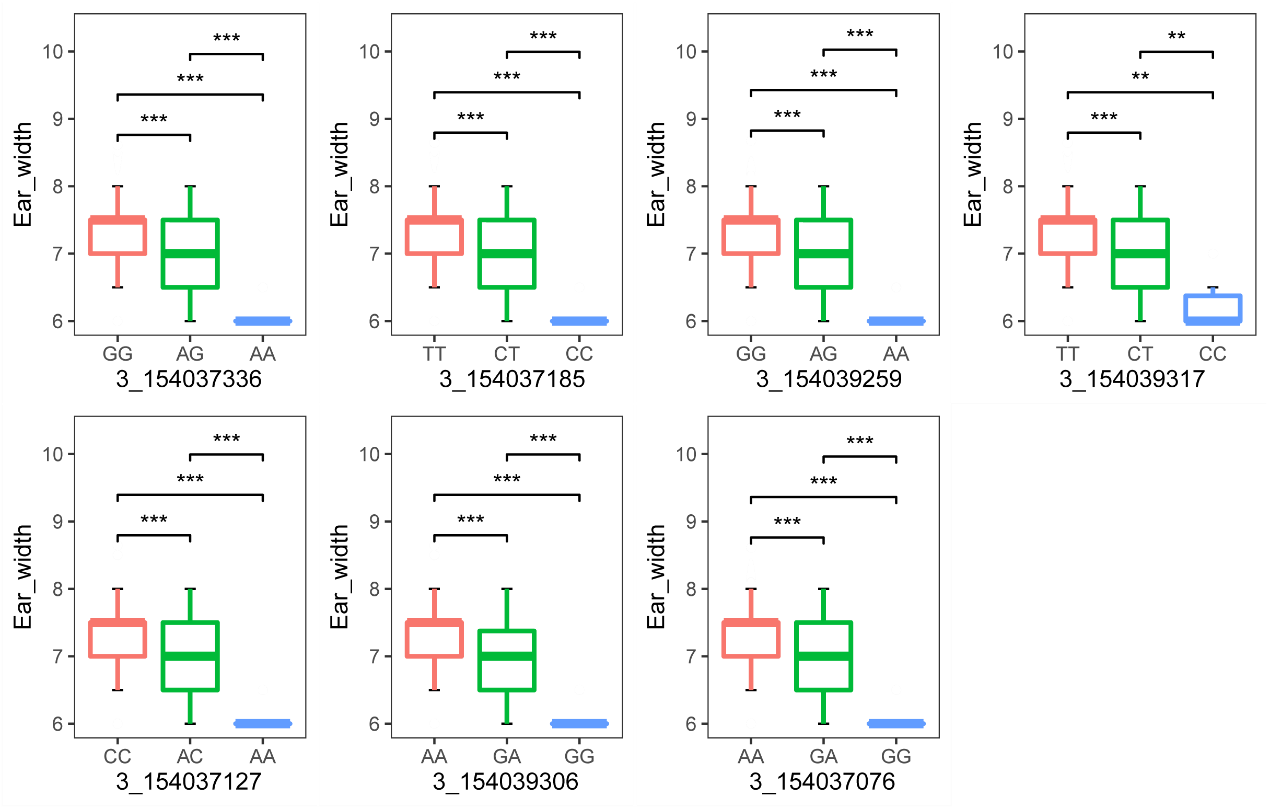

Supplement: S24 Fig — The x axial represents different genotypes, and the ordinate represents the ear width of the corresponding samples. The SNP on position chr3:154,039,306 is nonsynonymous. (TIF) [file pgen.1010615.s024.tif]

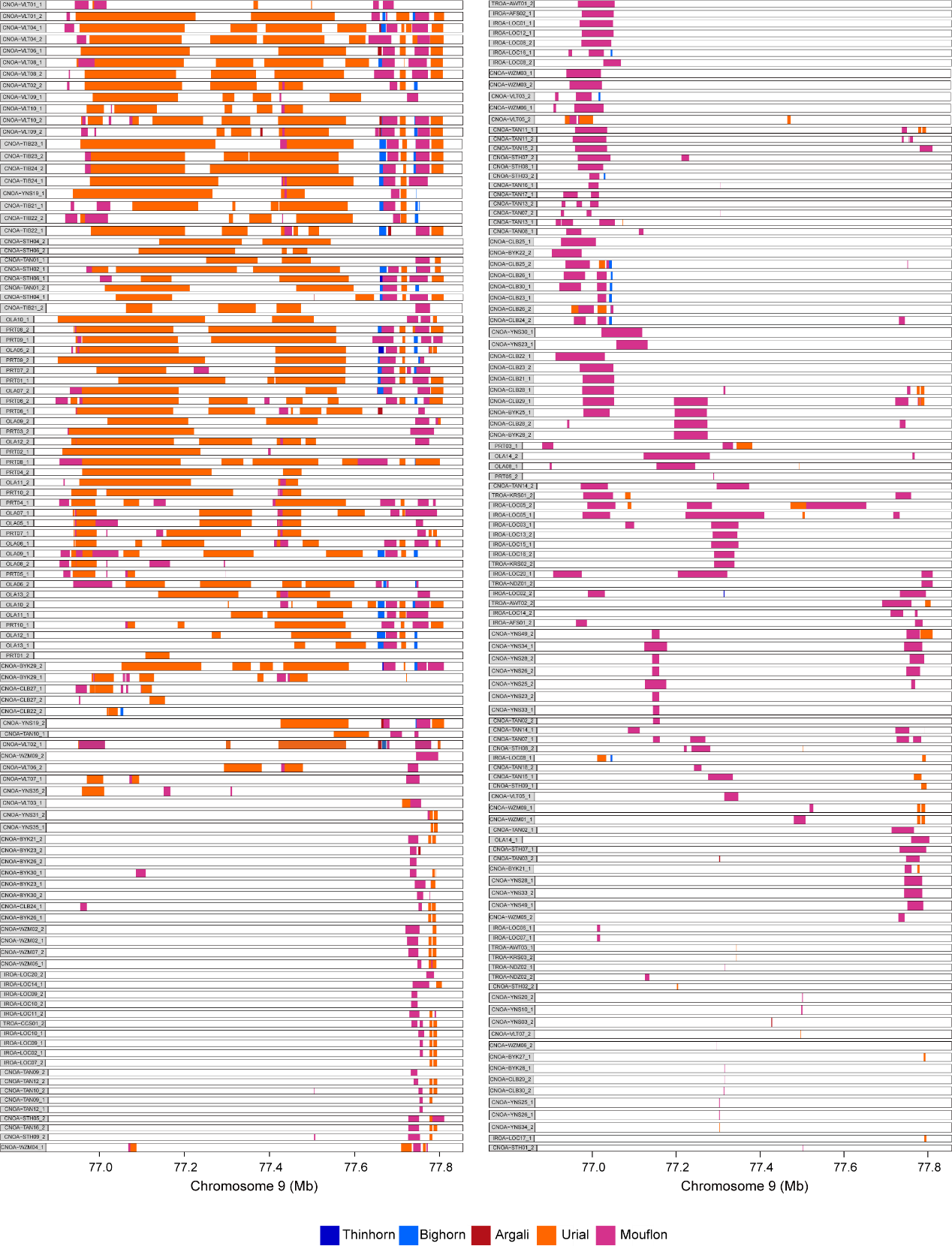

Supplement: S25 Fig — (TIF) [file pgen.1010615.s025.tif]

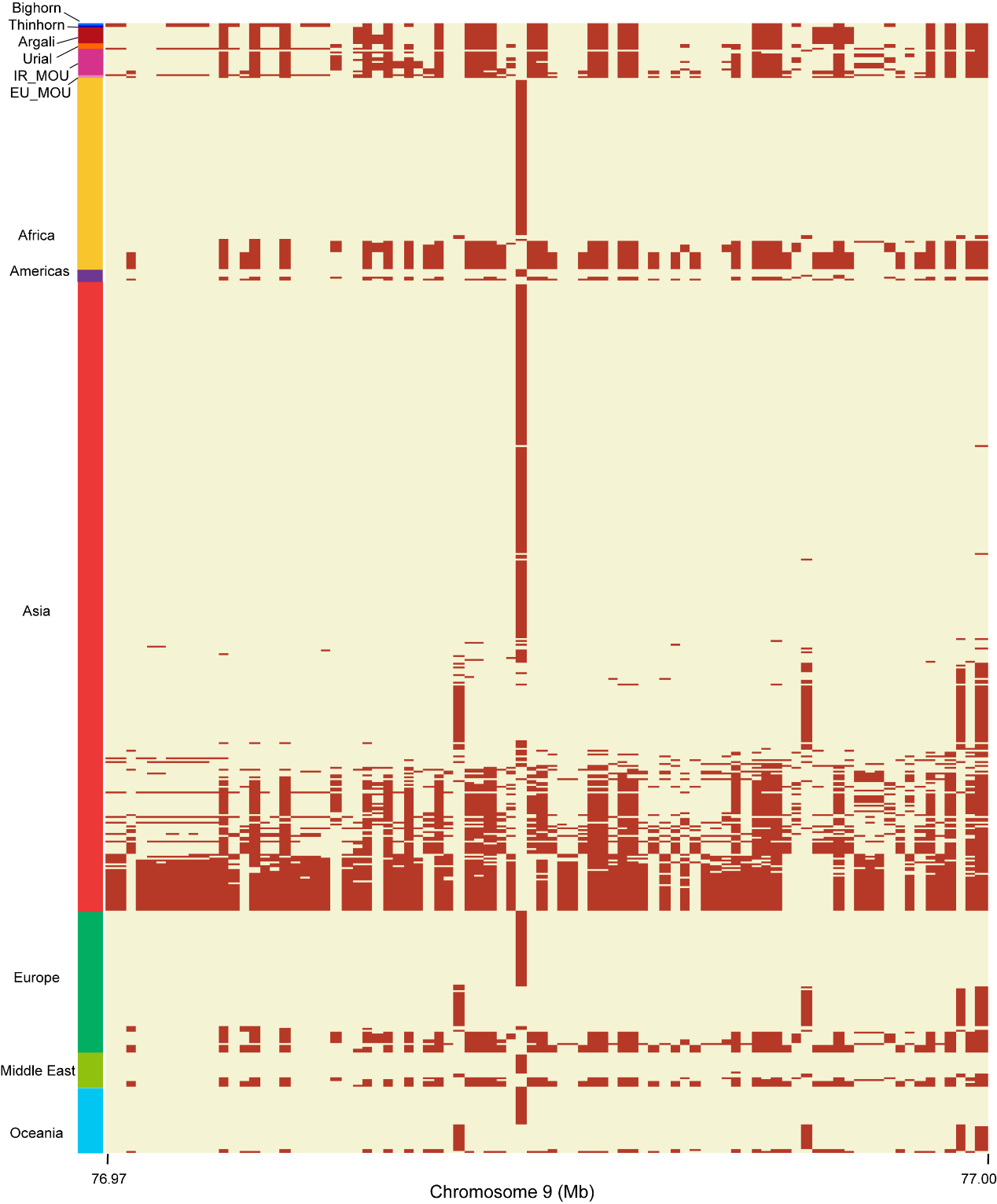

Supplement: S26 Fig — Each column represents a SNP variant, and each row represents a phased haplotype. The different color strips on the left show different groups. Yellow and red indicates the reference and the alternative alleles, respectively. (TIF) [file pgen.1010615.s026.tif]

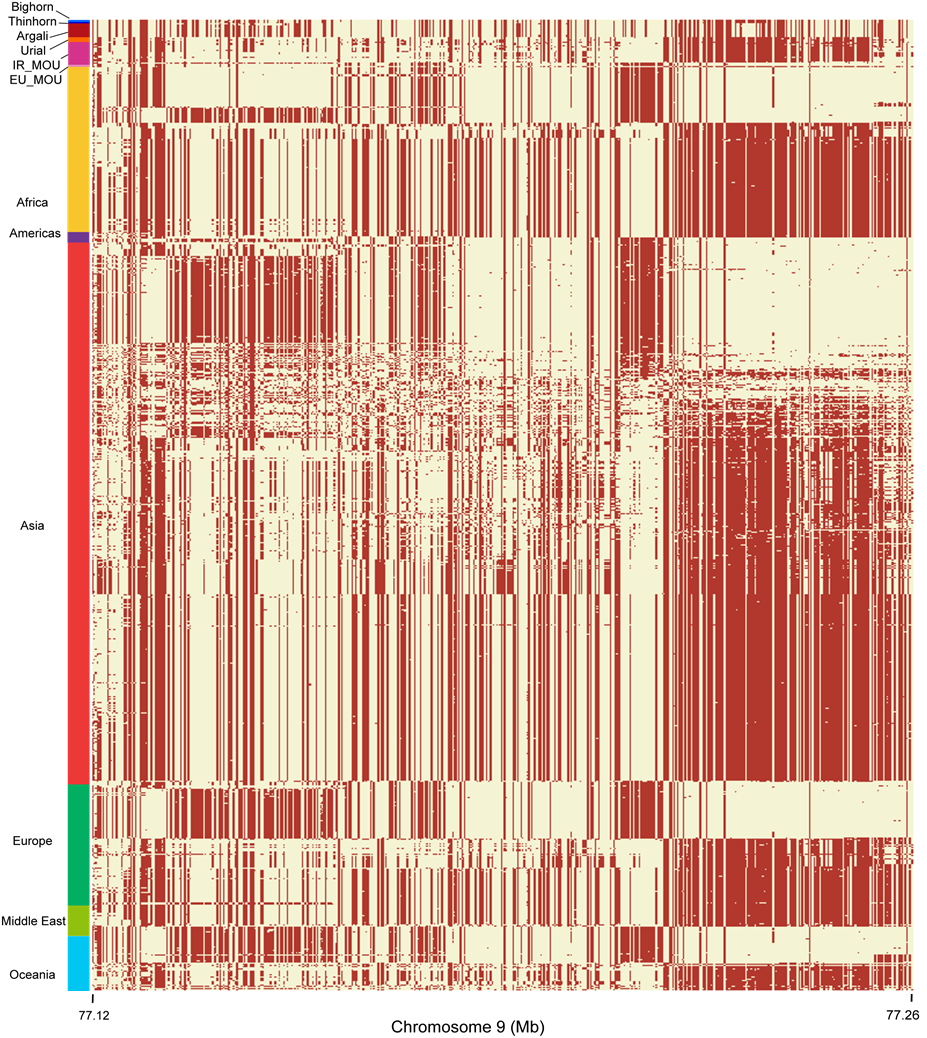

Supplement: S27 Fig — Each column represents a SNP variant, and each row represents a phased haplotype. The different color strips on the left show different groups. Yellow and red indicates the reference and the alternative alleles, respectively. (TIF) [file pgen.1010615.s027.tif]

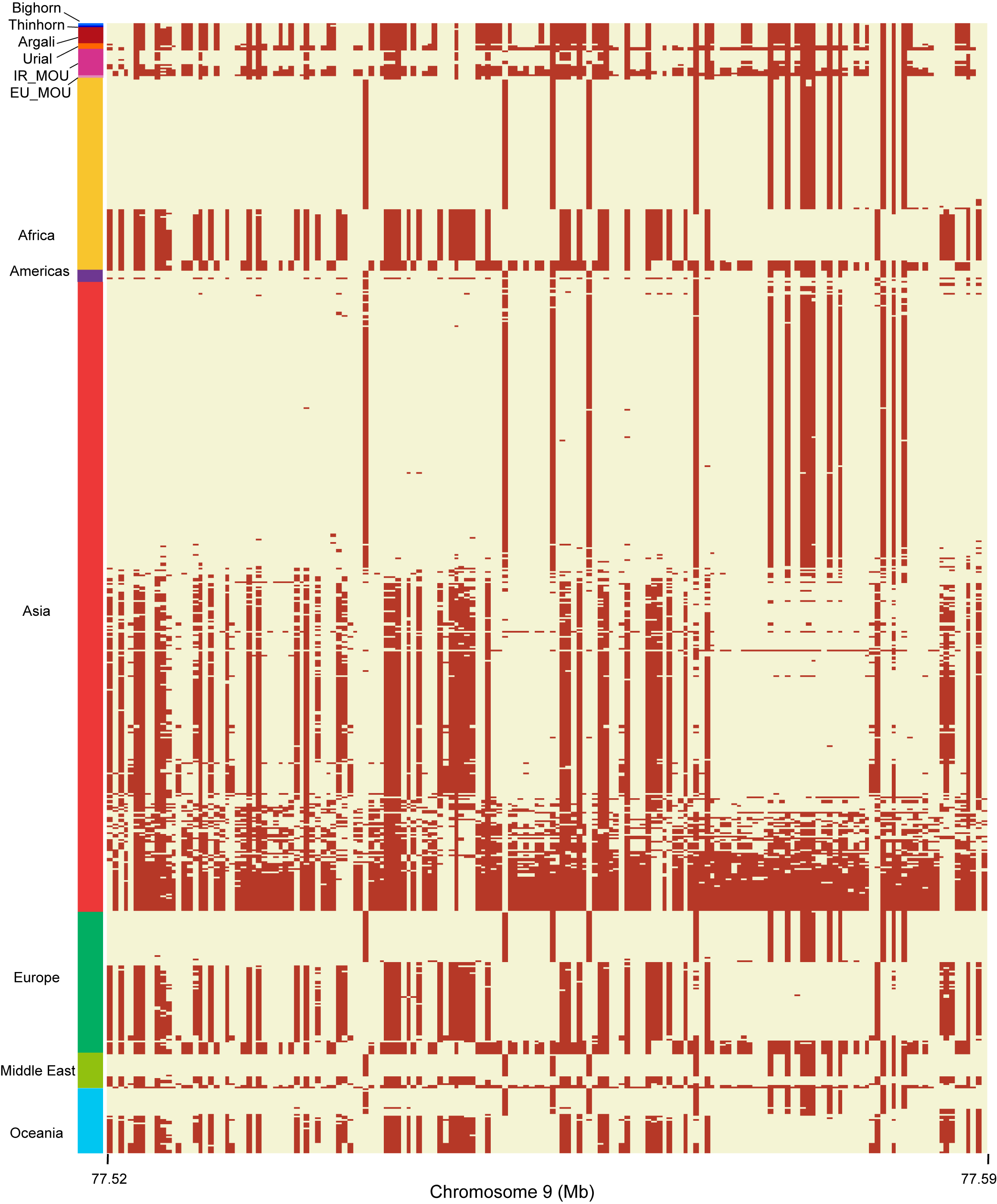

Supplement: S28 Fig — Each column represents a SNP variant, and each row represents a phased haplotype. The different color strips on the left show different groups. Yellow and red indicates the reference and the alternative alleles, respectively. (TIF) [file pgen.1010615.s028.tif]

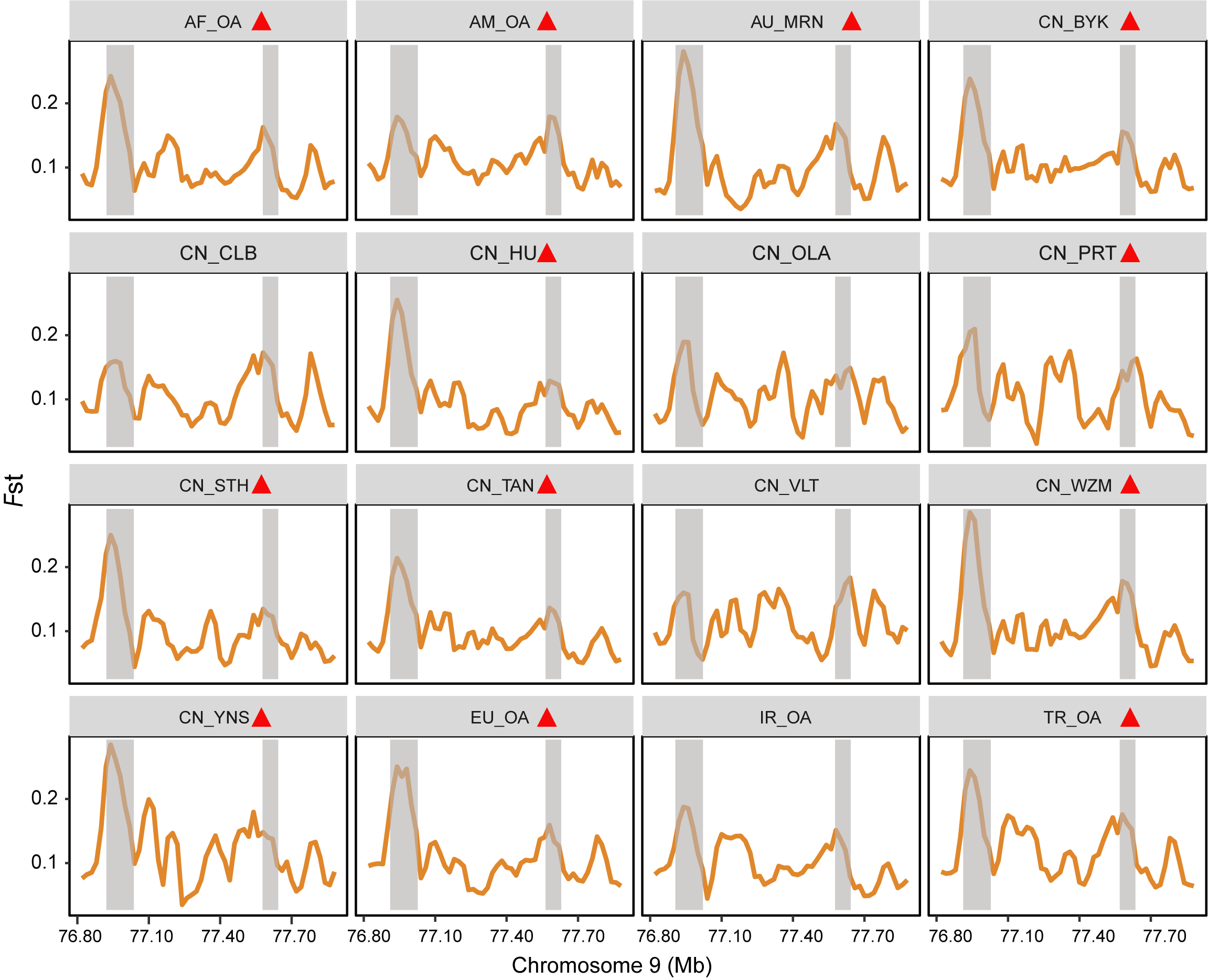

Supplement: S29 Fig — The FST was calculated in 50-kb sliding window with 20-kb step size. Triangles beside the population labels indicate that the population showed selective signals (FST top 1%) in VPS13B. The grey box means the location of windows showing selective signal. (TIF) [file pgen.1010615.s029.tif]

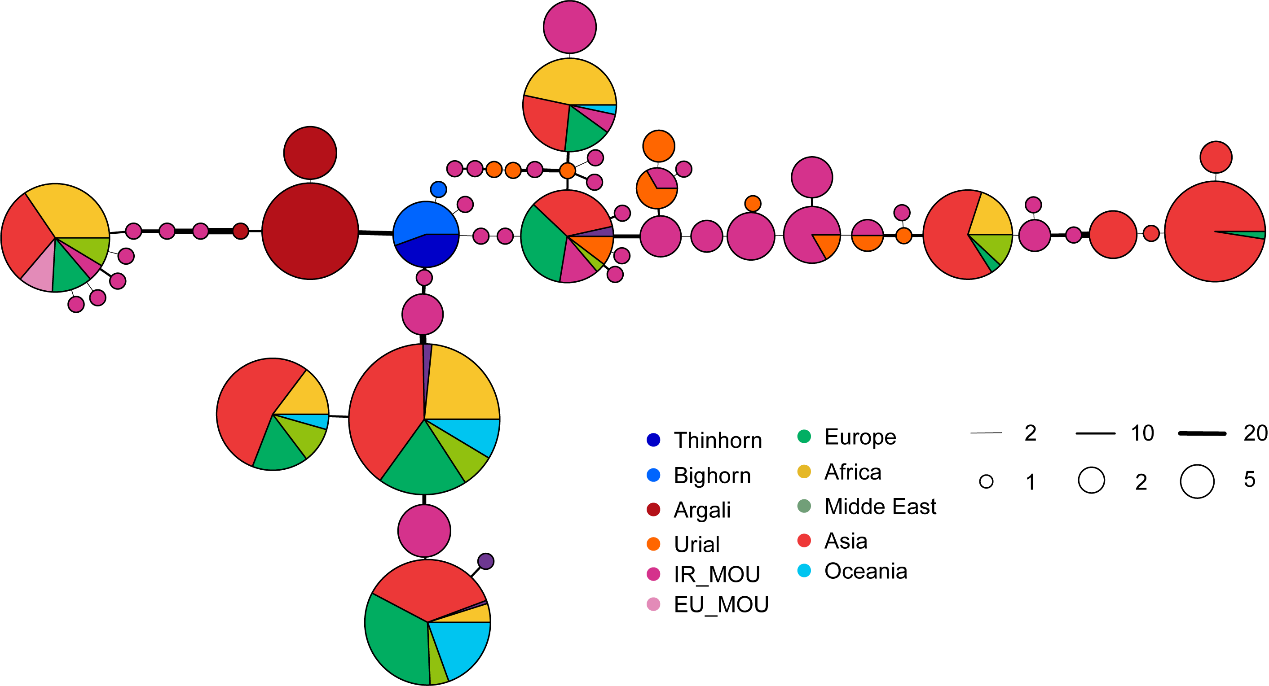

Supplement: S30 Fig — Different color shows different wild species or different regional sources of domestic sheep. The R software package PEGAS were used to generate the network. (TIF) [file pgen.1010615.s030.tif]

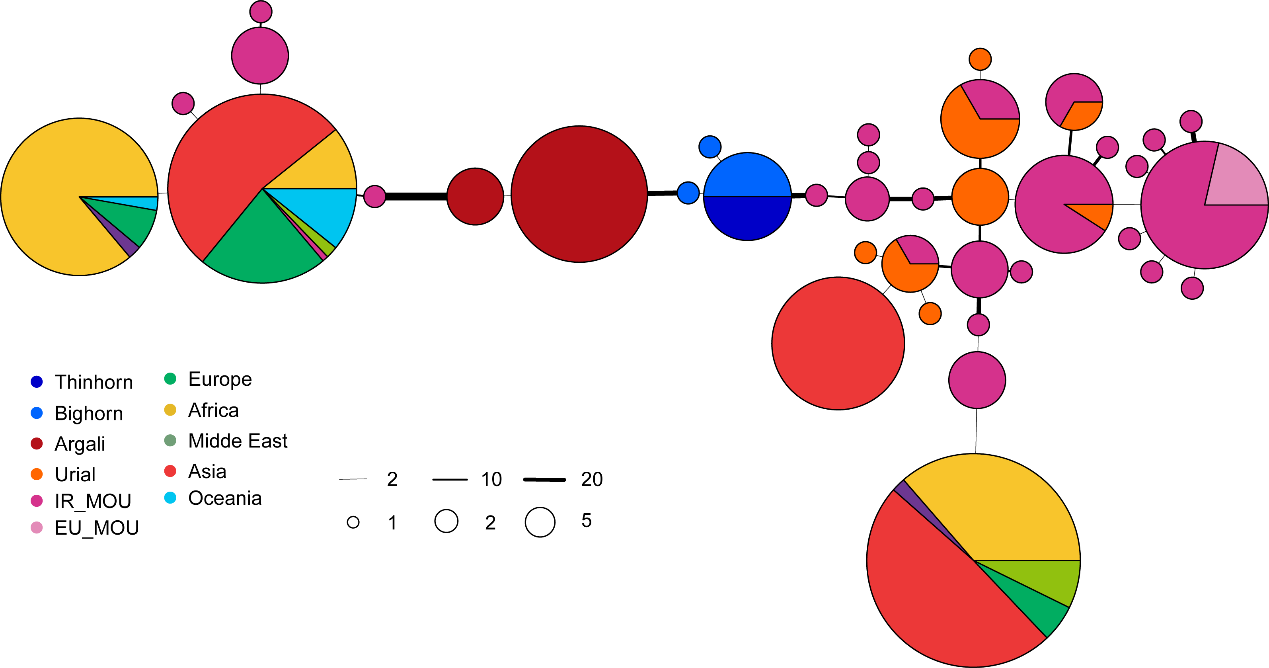

Supplement: S31 Fig — Different color shows different wild species or different regional sources of domestic sheep. The R software package PEGAS were used to generate the network. (TIF) [file pgen.1010615.s031.tif]

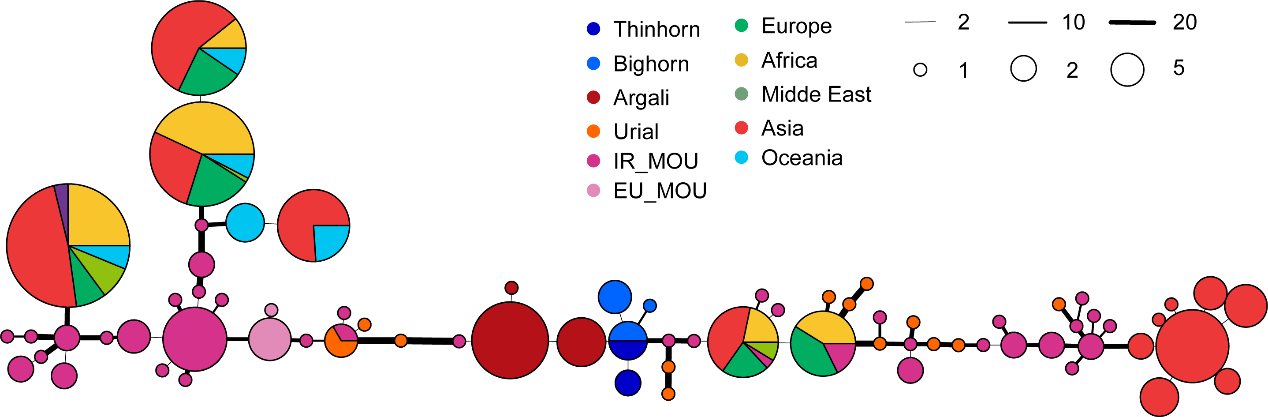

Supplement: S32 Fig — Different color shows different wild species or different regional sources of domestic sheep. The R software package PEGAS were used to generate the network. (TIF) [file pgen.1010615.s032.tif]
